# Blood Parasites in Domestic Birds in Central Iran

**DOI:** 10.3390/vetsci7030126

**Published:** 2020-09-04

**Authors:** Farzaneh Mirzaei, Abolghasem Siyadatpanah, Roghayeh Norouzi, Soheila Pournasir, Veeranoot Nissapatorn, Maria de Lourdes Pereira

**Affiliations:** 1 Department of Medical Parasitology and Mycology, School of Medicine, Shahid Sadoughi University of Medical Sciences, Yazd, Iran; mirzaei.farzaneh2015@yahoo.com (F.M.); Pornasir4126@gmail.com (S.P.); 2 Ferdows School of Paramedical and Health, Birjand University of Medical Sciences, Birjand, Iran; asiyadatpanah@yahoo.com; 3 Department of Pathobiology, Faculty of Veterinary Medicine, University of Tabriz, Tabriz, Iran; 4 School of Allied Health Sciences and Research Excellence Center for Innovation and Health Products (RECIHP), Walailak University, Nakhon Si Thammarat, Thailand; nissapat@gmail.com; 5 CICECO-Aveiro Institute of Materials & Department of Medical Sciences, University of Aveiro, 3810-193 Aveiro, Portugal

**Keywords:** blood parasites, central Iran, domestic birds, *Haemoproteus*, *Leucocytozoon*, *Plasmodium*

## Abstract

Parasites may affect the dynamics of bird populations. *Plasmodium*, *Leucocytozoon* and *Haemoproteus* are well-known avian haematozoa that can trigger decreased productivity and high mortality in domesticated birds. In this study, we evaluated the prevalence of avian blood parasites (*Plasmodium*, *Leucocytozoon* and *Haemoproteus*) against 335 birds of 8 species in the Yazd province in central Iran. To detect blood parasites, Giemsa-stained blood smears were prepared. Of the birds, 11.64% (39/335) were infected with at least one parasite genus, particularly *Haemoproteus* (32.6%; 23/335). The total prevalence values for *Plasmodium*, *Haemoproteus* and *Leucocytozoon* were 1.7, 6.8 and 2.9%, respectively. *Plasmodium* had lower prevalence rates of 1.7% (6/335). Among birds, pigeons, hens and ducks have the highest prevalence of *Haemoproteus*, *Leucocytozoon* and *Plasmodium* parasites at 1.7%, 6.8% and 2.9%, respectively. Results from this research extend our knowledge on the incidence of avian blood parasites in domesticated birds living in central Iran. The overall low incidence of avian blood parasites in birds was found in the Yazd province, Iran.

## 1. Introduction

The domestic poultry industry plays a very important role in providing a source of protein (meat and eggs) to humans, and in general plays a crucial role as a revenue provider in the national economy. Domestic poultry production in Iran is still distinctly divided into commercial and village business subsectors, each with its own particularities [1]. Different kinds of fungal, bacterial, viral, and parasitic pathogens can easily infest the domestic poultry. Parasitism ranks high among factors threatening the production of poultry. Haemoparasite infections are the most prevalent among the various parasitic diseases [2]. Haemosporidia blood parasites are vector-borne parasites that infect reptiles, birds, amphibians and mammals throughout the world [2]. Wild and domesticated birds are infected by a number of intracellular blood parasites, including *Plasmodium*, *Leucocytozoon* and *Haemoproteus*. Avian haemosporidians are a large group of protozoa, and approximately 200 species have been described [2,3,4]. Plasmodium is transmitted by mosquitoes (*Culicidae*), Haemoproteus is transmitted by biting midges (*Ceratopogonidae*), and Leucocytozoon is transmitted by black flies (*Simuliidae*) [2].

The effects of haemosporidian infections differ significantly, from no or just mild clinical impact to extreme morbidity or mortality between previously unexposed individuals [5]. Modern poultry farming has significantly reduced parasitic contamination, but this problem is still high in free-range breeding, reducing the bird’s normal activity and sometimes leading to death [6]. Other consequences of infection, such as decreased reproductive ability, coloring of plumage, impaired immune response, and increased aging may contribute to extinction of bird species. In addition, pathogens are commonly seen as regulators of the size of the host population [3,7].

The impact of changes associated with climate on the distribution and prevalence of vector-borne haemosporidian parasites is expected [8]. *Leucocytozoon*, *Plasmodium*, and *Haemoproteus* have been documented in various regions of the world, excluding Antarctica, where low temperatures do not enable the vectors to live [9]. Numerous studies have been performed in Iran focusing on the frequency, prevalence or incidence of blood parasites in birds, and indicate prevalence levels of 2–16% percent in various regions [10,11].

Taking into account the limited knowledge of blood parasitism among domestic birds in Iran, this study aimed to assess avian blood parasite (*Plasmodium*, *Leucocytozoon* and *Haemoproteus*) incidence in five areas of the Yazd Province, the central region of Iran.

## 2. Materials and Methods

### 2.1. Sample Collection

**Figure 1:**
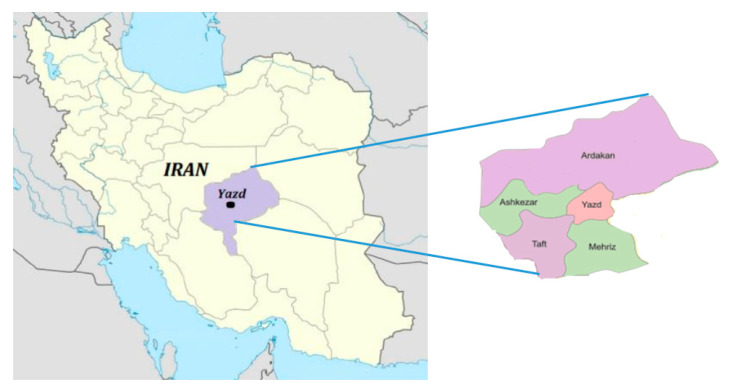
Map of sampling locations for birds in Yazd province in central Iran during 2018 and 2019.

This one-year study was carried out from February 2018 to January 2019 where we collected randomly 335 blood samples at five counties in Yazd province in central Iran (31°2′ N, 53°45′ E to 31°34′ N, 54°33′ E) (Figure 1). Sampling of male and female sexes of eight species of birds including hen (*Gallus gallus domesticus)* (*n* = 85), rooster (*Gallus domesticus)* (*n* = 30), pigeon (*Columba livia domestica)* (*n* = 75), quail (*Coturnix coturnix*) (*n* = 60), partridge (*Perdix perdix)* (*n* = 30), duck (*Bucephala albeola*) (*n* = 25), turkey (*Meleagris gallopavo)* (*n* = 23) and ostrich (*Struthio camelus)* (*n* = 7) was performed. Male and female birds were sampled equally. During the sampling, fabric rings were used around their tarsus to prevent repeated sampling of birds. Samples were obtained via brachial vein puncture of the birds with insulin needles and in tubes containing ethylenediaminetetraacetic acid (EDTA) as an anticoagulant for preparation of a thin blood smear.

### 2.2. Haemosporidian Parasite Detection

We prepared one thin smear of blood from each bird in the field. The smears were air dried, fixed with absolute (100%) methanol for 2 min, stained with Giemsa solution for 20 min, and diluted in buffer solution at 1:10 for 45 min. Finally, slides were washed gently under running tap water and air dried prior to microscopic assessment. Light microscope (Olympus microscope models manufacturer in japan 3H) was used to search for parasites in the blood smears. Two experienced microscopists examined the stained blood films blindly. In the event of a dispute between the two, all slides were recounted by them to reach a consensus. Subsequently, at least 100,000 erythrocytes (100 fields of microscopic) were examined in each smear [4], and the number of parasitized cells were observed microscopically at 1000× magnification using immersion oil.

### 2.3. Statistical Analysis

GraphPad PRISM v.5 (GraphPad Software, La Jolla, CA, USA; http://www.graphpad.com) was used to draw graphs and calculate the frequency.

## 3. Results

**Figure 2:**
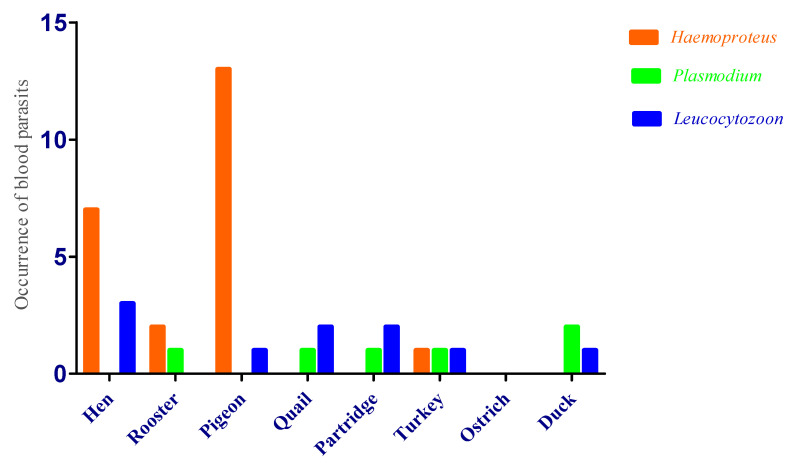
Estimated prevalence of blood parasites (*Haemoproteus, Plasmodium* and *Leucocytozoon*) infecting eight species of birds.

**Figure 3:**
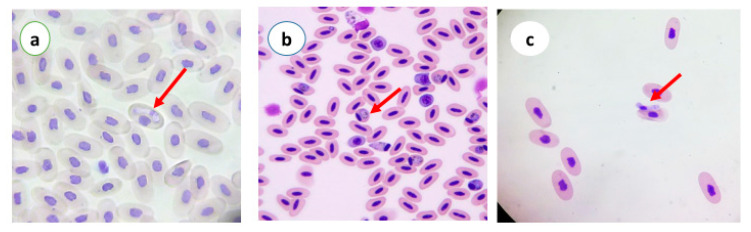
Blood smears of pigeon stained with Giemsa: (**a**) *Haemoproteus (*×1000); *(***b**) *Plasmodium* (×400); and (**c**) *Leucocytozoon* (×400).

A total of 335 birds belonging to 5 counties in the Yazd Province in central Iran were analyzed (Table 1, Table 2 and Table 3). Of the birds, 11.64% (39/335) showed at least one parasite specimen of *Haemoproteus, Leucocytozoon*, or *Plasmodium*. The overall incidence values for *Plasmodium*, *Haemoproteus* and *Leucocytozoon* were 1.7%, 6.8% and 2.9% respectively. The rates of parasite prevalence among bird families were not homogeneous. *Plasmodium* prevalence was low as compared to *Haemoproteus,* and *Leucocytozoon* was observed in six of eight bird species (Figure 2). Pigeon was the bird species with the highest *Haemoproteus* prevalence (18.8%), while quail, partridge, ostrich and duck were not infected with *Haemoproteus*. *Plasmodium* was identified in five species: rooster (*n* = 1; 1.1%), quail (*n* = 1; 1.4%), partridge (*n* = 1; 1.4%), turkey (*n* = 1; 1.4%) and duck (*n* = 2; 3.1%) (Table 2). Hen was the bird species with the highest *Leucocytozoon* prevalence (4.3%), while rooster was not infected with *Leucocytozoon*. *Leucocytozoon* was identified in five species: pigeon (*n* = 1; 1.1%), quail (*n* = 2; 2.8%), partridge (*n* = 2; 3.1%), turkey (*n* = 1; 1.1%) and duck (*n* = 1; 1.4%) (Table 3). The ostrich was not infected with any blood parasites. The number of male and female birds was the same, but we found no relationship between the sex of the birds and their incidence of blood parasites. The blood parasite (*Haemoproteus*, *Plasmodium* and *Leucocytozoon*) images are shown in Figure 3. Mix infection was not observed in this study.

## 4. Discussion

Avian haematozoa parasites may induce declining productivity and a high death rate in domestic birds. Birds from most Neotropical areas appear to have low blood parasite prevalence rates, regardless of habitat or region type [12]. Our study investigated the incidence of avian blood parasites (*Haemoproteus*, *Leucocytozoon* and *Plasmodium*) between eight species of birds located in Yazd province in central Iran. In our study, 11.64% of the 335 birds sampled showed a general blood parasite prevalence. The total prevalence values for *Plasmodium*, *Haemoproteus* and *Leucocytozoon* were 1.7%, 6.8% and 2.9% respectively. *Plasmodium* had lower prevalence rates (1.7%; 6/335). Among the birds, pigeons, hens and ducks have the highest prevalence of *Haemoproteus*, *Leucocytozoon* and *Plasmodium* parasites at 1.7%, 6.8% and 2.9%, respectively. In this study, pigeons were more infected with *Haemoproteus* than other blood parasites, which is due to the high population of vector (biting midges) in their habitat and low use of insecticides.

Different studies have yielded different results, and these variations have not been clarified for reasons, but may include the season of the year during which blood samples were collected, the ecological and behavioral characteristics of the species, regional climate, habitat-dependent vector distribution, host species and age structure [7,13]. Unfortunately, in this study, sampling of birds was not performed uniformly in each season, so we could not find a relationship between the prevalence of blood parasites and different seasons, which is one of the limitations of this study.

Previous reports have suggested that low blood parasitemia rates provide some defense against infection [14]. Some birds may therefore show no signs of illness but maintain infection, enabling parasites to survive in the dry season when vector populations are low. In our study, due to the warm and dry climate of Yazd province, the prevalence of blood parasites was low. However, there is little information regarding the interaction between the parasite and the host is complex. Among the studied birds, quail, partridge, ostrich and duck were not infected with the *Haemoproteus* parasite, which does not depend on factors such as climate and humidity, because the characteristics of our study sites were generally similar in terms of climate and humidity. On the other hand, host characteristics such as sex was not related to infection status, but this may be explained in part by the ecology of the bird species and their immunity. Among the five cities of Yazd province, the highest rate of blood parasites was observed in Taft city, which could be due to the low level of health and low use of insecticides and high population of vectors in this city when compared to other cities.

Studies from different countries such as Brazil show varying prevalence of blood parasites (15.8% among 925 birds) [7]. Blood parasite prevalence of chickens and turkeys in a study by Opara et al. [6] in Nigeria showed 12%, with 8.9% for chickens and 40% for turkeys. The results of this study are less than results from a study in Ghana [15], which observed 27% prevalence, while 71% prevalence was observed in Malawi [16] and 61.9% in Uganda [17]. Hussein and Abdelrahim. [18] histopathologically examined the liver and lungs of 103 pigeons, and their study showed different stages of *Haemoproteus columbae* in the blood, liver and lungs of the pigeons captured with high prevalence (57.2%) [18]. The prevalence of *Haemoproteus columbae* in different areas of Mymensingh district of Bangladesh was 20% [19]. In India, the prevalence for *Haemoproteus* was 18% [20].

In a study of 72 blood samples from 26 chickens, 24 ducks and 22 pigeons, Momin et al. [21] concluded that 33 (45.8%) of the samples examined were found to be infected with various protozoa in the blood. Two species of blood protozoa were identified, namely, *Leucocytozoon* spp. in chickens (34.6%) and ducks (58.3%), and *Haemoproteus* spp. (22.7%) and *Leucocytozoon* spp. (22.7%) in pigeons [21].

Molecular studies such as PCR are reliable methods for assessing the prevalence, species identification and phylogenetic of parasites, especially in the case of blood parasites that have periodicity. Tabaripour et al. [22] investigated the molecular and structural properties of *Haemoproteus* protozoa by PCR in 120 blood samples of infected pigeons in Mazandaran province, Iran. The results showed that 17 samples were positive, indicating an infection rate of 11.33% [22]. In our study, the infection rate of pigeons with *Haemoproteus* protozoa was 17.3%, which is probably due to the difference in climate between the two provinces and the activity of the carriers. The climate of Mazandaran province is hot and humid, but the climate of Yazd province is hot and dry.

## 5. Conclusions

The findings of this study have shown that infection with haemoparasites is less frequent in farm poultry birds in the Yazd province, Iran. Further studies must address the effect of different seasons of the year and parasitic burden. Although the percentage of parasites reported in the present investigation is low, possible implications for human health through the consumption of some of these species may be a concern.

## Figures and Tables

**Table 1:** Occurrence of *Haemoproteus* (Haem) parasite in eight species of birds located at five counties in Yazd province, central Iran.

Location/Bird Types	No.	Occurrence of *Haemoproteus* (*Haem*) Parasite								Total
		Hen	Rooster	Pigeon	Quail	Partridge	Turkey	Ostrich	Duck	
**Yazd**	55	1 (1.8)	0	1 (1.8)	0	0	0	0	0	2 (3.6)
**Mehriz**	86	1 (1.1)	1 (1.1)	3 (3.5)	0	0	1 (1.1)	0	0	6 (6.9)
**Taft**	70	3 (4.3)	1 (1.4)	5 (7.1)	0	0	0	0	0	9 (12.8)
**Ashkezar**	68	2 (2.9)	0	2 (2.9)	0	0	0	0	0	4 (5.8)
**Ardakan**	56	0	0	2 (3.5)	0	0	0	0	0	2 (3.5)
**Total**	355	7 (10.1)	2 (2.5)	13 (18.8)	0	0	1 (1.1)	0	0	23 (32.6)

**Table 2:** Occurrence of *Plasmodium* (Plas) parasite in eight species of birds located at five counties in Yazd province, central Iran.

Location/Bird Types	No.	Occurrence of *Plasmodium (Plas)* Parasite								Total
		Hen	Rooster	Pigeon	Quail	Partridge	Turkey	Ostrich	Duck	
**Yazd**	55	0	0	0	0	0	0	0	0	0
**Mehriz**	86	0	1 (1.1)	0	0	0	0	0	0	1 (1.1)
**Taft**	70	0	0	0	1 (1.4)	1 (1.4)	1 (1.4)	0	0	3 (4.2)
**Ashkezar**	68	0	0	0	0	0	0	0	1 (1.4)	1 (1.4)
**Ardakan**	56	0	0	0	0	0	0	0	1 (1.7)	1 (1.7)
**Total**	355	0	1 (1.1)	0	1 (1.4)	1 (1.4)	1 (1.4)	0	2 (3.1)	6 (8.4)

**Table 3:** Occurrence of *Leucocytozoon* (*Leuc*) parasite in eight species of birds located at five counties in Yazd province, central Iran.

Location/Bird Types	No.	Occurrence of *Leucocytozoon (Leuc)* Parasite								Total
		Hen	Rooster	Pigeon	Quail	Partridge	Turkey	Ostrich	Duck	
**Yazd**	55	1 (1.8)	0	0	0	0	0	0	0	1 (1.8)
**Mehriz**	86	1 (1.1)	0	1 (1.1)	0	0	1 (1.1)	0	0	3 (3.3)
**Taft**	70	1 (1.4)	0	0	1 (1.4)	0	0	0	0	2 (2.8)
**Ashkezar**	68	0	0	0	1 (1.4)	1 (1.4)	0	0	1 (1.4)	3 (4.2)
**Ardakan**	56	0	0	0	0	1 (1.7)	0	0	0	1 (1.7)
**Total**	355	3 (4.3)	0	1 (1.1)	2 (2.8)	2 (3.1)	1 (1.1)	0	1 (1.4)	10 (13.8)

## References

[1] Nnadi P., George S. (2010). A cross-sectional survey on parasites of chickens in selected villages in the subhumid zones of South-Eastern Nigeria. J. Parasitol. Res..

[2] Valkiunas G. (2004). Avian Malaria Parasites and Other Haemosporidia.

[3] Zamora-Vilchis I., Williams S.E., Johnson C.N. (2012). Environmental temperature affects prevalence of blood parasites of birds on an elevation gradient: Implications for disease in a warming climate. PLoS ONE.

[4] Atkinson C.T., Thomas N.J., Hunter D.B. (2009). Parasitic Diseases of Wild Birds.

[5] Dimitrov D., Palinauskas V., Iezhova T.A., Bernotienė R., Ilgūnas M., Bukauskaitė D., Zehtindjiev P., Ilieva M., Shapoval A.P., Bolshakov C.V. (2015). Plasmodium spp.: An experimental study on vertebrate host susceptibility to avian malaria. Exp. Parasitol..

[6] Opara M., Osowa D., Maxwell J. (2014). Blood and gastrointestinal parasites of chickens and turkeys reared in the tropical rainforest zone of southeastern Nigeria. Open J. Vet. Med..

[7] Sebaio F., Braga É.M., Branquinho F., Fecchio A., Marini M.Â. (2012). Blood parasites in passerine birds from the Brazilian Atlantic Forest. Rev. Bras. Parasitol. Veterinária.

[8] Van Hemert C., Meixell B.W., Smith M.M., Handel C.M. (2019). Prevalence and diversity of avian blood parasites in a resident northern passerine. Parasit. Vectors.

[9] Friend M., Franson J.C., Ciganovich E.A. (1999). Field Manual of Wildlife Diseases: General Field Procedures and Diseases of Birds.

[10] Borji H., Moghaddas E., Razmi G., Bami M.H., Mohri M., Azad M. (2011). Prevalence of pigeon haemosporidians and effect of infection on biochemical factors in Iran. J. Parasit. Dis..

[11] Dezfoulian O., Zibaei M., Nayebzadeh H., Haghgoo M., Emami-Razavi A., Kiani K. (2013). Leucocytozoonosis in domestic birds in southwestern Iran: An ultrastructural study. Iran. J. Parasitol..

[12] Fecchio A., Marini M.Â., Braga É.M. (2007). Baixa prevalência de hemoparasitos em aves silvestres no Cerrado do Brasil Central. Neotrop. Biol. Conserv..

[13] Fecchio A., Lima M.R., Silveira P., Braga É.M., Marini M.Â. (2011). High prevalence of blood parasites in social birds from a neotropical savanna in Brazil. Emu-Austral Ornithol..

[14] Gong L., Maiteki-Sebuguzi C., Rosenthal P.J., Hubbard A.E., Drakeley C.J., Dorsey G., Greenhouse B. (2012). Evidence for both innate and acquired mechanisms of protection from *Plasmodium falciparum* in children with sickle cell trait. Blood.

[15] Poulsen J., Permin A., Hindsbo O., Yelifari L., Nansen P., Bloch P. (2000). Prevalence and distribution of gastro-intestinal helminths and haemoparasites in young scavenging chickens in upper eastern region of Ghana, West Africa. Prev. Vet. Med..

[16] Njunga G.R. (2003). Ecto-and Haemoparasites of Chickens in Malawi with Emphasis on the Effects of the Chicken Louse, Menacanthus Cornutus. Unpublished. Master’s Thesis.

[17] Valkiūnas G., Sehgal R.N., Iezhova T.A., Smith T.B. (2005). Further observations on the blood parasites of birds in Uganda. J. Wildl. Dis..

[18] Hussein N., Abdelrahim E. (2016). Haemoproteus columbae infection and its histopathological effects on pigeons in Qena Governorate, Egypt. J. Pharm. Biol. Sci. (IOSRJPBS).

[19] Dey A., Begum N., Anisuzzaman A., Khan M., Mondal M. (2008). Haemoprotozoan infection in ducks: Prevalence and pathology. Bangladesh J. Vet. Med..

[20] SENLIK B., GULEGEN E., AKYOL V. (2005). Prevalance and intensity of haemoproteus columbae in domestic pigeons. Indian Vet. J..

[21] Momin M.A., Begum N., Dey A.R., Paran M.S., Alam M.Z. (2014). Prevalence of blood protozoa in poultry in Tangail, Bangladesh. IOSR J. Agric. Vet. Sci..

[22] Tabaripour R., Youssefi M., Rahbari S., Arghavan M. (2017). Molecular identification of Haemoproteus in domestic pigeons (Colombia livia domestica) in Mazandaran province. J. Vet. Res..

